# Ke Hsin Kuo: A distinguished scientist and great mentor

**DOI:** 10.1007/s13238-018-0589-5

**Published:** 2018-11-13

**Authors:** Da-Neng Wang, Lu-Chang Qin

**Affiliations:** 1 Skirball Institute of Biomolecular Medicine, and Department of Cell Biology, New York University School of Medicine, 540 First Avenue, New York, NY 10016 USA; 2 W.M. Keck Laboratory for Atomic Imaging and Manipulation, Department of Physics and Astronomy, University of North Carolina, Chapel Hill, NC 27599 USA

## Early years

Professor Ke Hsin Kuo was born in Beijing in 1923, and his childhood was spent in Harbin in northeastern China where his father worked as an engineer to build the local railroad. His early education was at best characterized by frequent disruptions, the first of which being the “9.18 Incident” of 1931 when the Japanese Kwantung Army launched an assault in Shenyang (Mukden) on the local Chinese troops and quickly took control of all the Northeastern Provinces (Manchuria). He moved to Tianjin in the spring of 1936, where he continued his education by enrolling in the famous Nankai High School. However, his school life at Nankai was soon disrupted again by the eruption of full-scale war in July of 1937 after Japan attacked and occupied Peking. Together with his brothers, he fled to join his parents who had previously relocated to Chongqing. He studied in Chongqing for four more years to complete his high school education, during which classes were constantly being disrupted due to the frequent bombings of the city by Japanese airplanes. These bombings not only left him with long lasting memories of the war, but also bore him a permanent burn scar on his back as a result of a bomb that exploded just meters away from him.

In 1941 Professor Kuo entered Zhejiang University, a national university that was evacuated to Guizhou Province from Hangzhou due to the advancement of the Japanese invasion. He graduated in 1946 having majored in Chemical Engineering. When an opportunity arose after the war ended in 1945, he took a competitive national examination and won a government scholarship to study metallurgy in Sweden.

**Figure 1:**
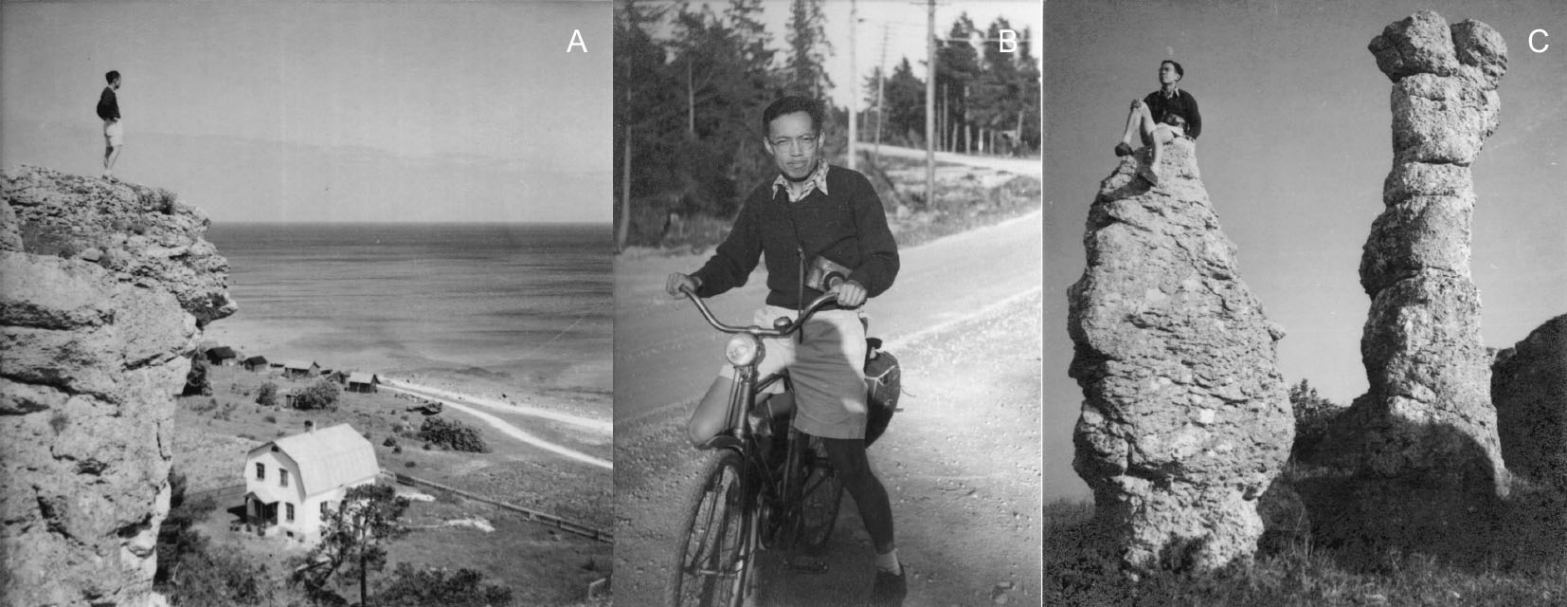
**In Sweden in early 1950s (Photographs courtesy of Ms. Hua Guo)**

Professor Kuo arrived in Stockholm in September 1947 to start his study of metallography with Professor Axel Hultgren at the Royal Institute of Technology (KTH) (Fig. 1). While actively engaged in research in traditional metallography, he also became increasingly interested in the newly developed X-ray diffraction methods. Later in 1950, after learning more about the usefulness and power of X-ray diffraction and crystallography in microstructural research of alloys, he decided to abandon his almost-completed doctoral thesis at KTH (although he eventually returned in 1980 to accept an honorary doctoral degree together with Foreign Membership in the Royal Swedish Academy of Engineering), and moved to Uppsala University. There he pursued X-ray diffraction studies of carbides in alloys in the Department of Inorganic Chemistry with Professor Gunnar Hägg, a world leading figure in X-ray crystallography at the time. With Hägg he soon published his first research paper in the journal *Nature* (Kuo and Hägg, 1952). He studied more structures of carbides, including η carbide (Kuo, 1953), which has a crystal structure closely related to that of a quasicrystal which would be discovered many years later in his laboratory in China. He returned to KTH briefly in 1954, where he carried out and published his first piece of work using another new technique—electron microscopy—for characterization of carbide precipitates in alloys (Kuo, 1956).

Professor Kuo briefly went to the Delft Institute of Technology in the Netherlands at the end of 1955 to work with Professor W.G. Burgers, to study the phase transformation of white tin to grey tin. In addition to his work on carbides, he also published several research papers on intermetallic phases including the structure of the Laves and σ phases, which would all be closely related to his work decades later on quasicrystals.

Professor Kuo was now well equipped with the knowledge and expertise in metallurgy, X-ray crystallography and electron microscopy needed to start his own lab. In April 1956, he left Europe after staying there for nine years in response to a call by the then Premier Zhou Enlai for overseas students to return to their homeland to build a new China.

## Electron diffraction and microscopy for materials research in China

Upon his return to China, Professor Kuo was already an established physical metallurgist in his own right—he had more than twenty published papers to his credit. He joined the newly established Institute of Metal Research (IMR), Chinese Academy of Sciences, in Shenyang as a full professor. Bearing high hopes for using his expertise to contribute to the development of China, he accepted his very first graduate student in 1956 and began to assemble a capable research team. Despite a series of political turmoils that escalated one after another in China at this time, he did his best to keep his research going. His laboratory acquired a modern transmission electron microscope (TEM, JEM-150) in 1965 that allowed them to perform electron diffraction as well as diffraction contrast imaging of crystal defects, a technique developed only in the early 1960’s. With this TEM he and his associates studied crystal defects in Ni-based alloys. However, their work did not continue unabated for long as the next political vagary, the “Cultural Revolution”, started to hamper his research in 1966. During the ten years of the “Cultural Revolution” until it formally ended in 1977, his connections to the outside world were essentially severed, and all of his basic research and technical teaching came to a complete standstill. As a result, Professor Kuo did not begin publishing papers again in international journals until 1980. However, even during the darkest times he never gave up his dream of again applying his expertise and skills to advance science in China and, with whatever means available, he grasped every possible opportunity to refresh his knowledge and train young scientists. For instance, in the mid-1970s, as soon as the political environment began to relax a little, he organized “personal” workshops or study groups to teach the latest developments in materials science to his junior colleagues, attesting to his personal resilience and intellectual stamina. A highly valued and demanded outcome of these endeavors was his personal notebook on electron diffraction, which was widely hand-copied as a high-level technical treatise within his small community.

Starting in 1978, Professor Kuo was once again able to accept graduate students into his lab, and he quickly instituted a research program in materials science using electron diffraction and microscopy techniques. Using the JEM-150 microscope equipped with an *in situ* heating stage, he directed his team to study the microstructural evolution during crystallization of various Ni-based metallic glasses. His group also systematically analyzed the use of electron diffraction geometry and initiated the use of computers to develop an automated program for indexing of electron diffraction patterns in China. As a result, he and his colleagues published a book on electron diffraction in 1983, which has trained and benefited generations of students and users throughout the country (Kuo et al., 1983).

The rapid development of high-resolution electron microscopy (HREM) in the late 1970s caught Professor Kuo’s attention. With the re-establishment of connections with the outside world allowing him to learn the latest developments in electron microscopy, especially stimulated by intimate discussions with two high-level visiting delegations of electron microscopists from Japan including Drs. Hatsujiro Hashimoto, Sumio Iijima and Ryozi Uyeda, Professor Kuo soon chose the characterization of solid state materials using HREM as the major research area for his revitalized laboratory.

Professor Kuo attracted a number of talented investigators into his lab. Recognizing the time lost to the upheavals of the past decades and his programs’ lack of expertise in recently developed techniques, he worked tirelessly himself and also sent his junior associates abroad to top labs to obtain advanced training and experience. These efforts reaped noticeable benefits and quick rewards to his research program. Beginning in late 1982, with the acquisition of a brand new JEM 200CX TEM, one of the first installed in China, his team started applying HREM to various studies including defect analysis of alloys, metastable phase transformation in metallic glasses, semiconductors, and catalysts, complemented with image simulations and processing, illustrating his eclectic taste in research topics. He also organized advanced workshops and invited prominent scientists from around the world to lecture in China and to interact with the members and graduate students in his group. To advance electron microscopy for materials research, Professor Kuo established and served as the founding director of the Laboratory for Atomic Imaging of Solids at IMR, a dedicated electron microscopy laboratory that to this day continues to make significant contributions to the science of electron microscopy. In 1985, he also founded the Beijing Laboratory of Electron Microscopy (BLEM), Chinese Academy of Sciences. Due to his significant contributions to science, in 1980 Professor Kuo was elected as a member of the Chinese Academy of Sciences and the Royal Swedish Academy of Engineering,

## Quasicrystal research

Given his broad knowledge and deep understanding of metallic structures and the intensive effort he devoted to the study of the phase transitions of metallic glasses through a series of metastable phases, it came as no surprise, that Professor Kuo’s team quickly made many discoveries, particularly in quasicrystal research. In intermetallic compounds, metal atoms often form clusters with slightly distorted symmetry from ideal icosahedral packing, such as the Laves, σ, µ, H and C phases, collectively called the Frank-Kasper phases. In these structures, the individual structural unit is actually composed of a distorted icosahedral cluster (pentagonal antiprism) arranged into a column along a five-fold axis. As a result, in a heavily faulted structure of these phases, the electron diffraction pattern will instead exhibit more characteristics of the individual topological unit, and distinct features reflecting the structure of icosahedral clusters, i.e., five-fold symmetry, should appear. Indeed, it was soon observed in heavily faulted alloys with nano-domains of the Frank-Kasper phases that an electron diffraction pattern with apparent ten-fold symmetry was present (Ye et al., 1984). In another investigation conducted in parallel examining the crystallization of metallic glasses in a (Ti_0.9_V_0.1_)_2_Ni alloy, Ze Zhang, his doctoral student who at the time worked with him and Professor Heng-Qiang Ye, soon obtained electron diffraction patterns displaying icosahedral symmetry and high resolution TEM images of the samples that directly resembled quasi-periodic Penrose tiling. This work announced the discovery of quasicrystals in the Ni-Ti alloy, a totally new metallic system (Zhang et al., 1985). In the meantime, Professor Kuo’s team also demonstrated side by side the fundamental differences in atomic structure from five-fold twinning that could result in an electron diffraction pattern with apparent ten-fold symmetry (Jiang et al., 1985). The entire project was carried out independently from the results published in *Physical Review Letters* only a few months earlier reporting the identification of icosahedral quasicrystals in an Al-Mn alloy with five-fold symmetry that is “forbidden” in classical crystallography (Shechtman et al., 1984), a discovery that revolutionized crystallography and later won the 2011 Nobel Prize in Chemistry.

**Figure 2:**
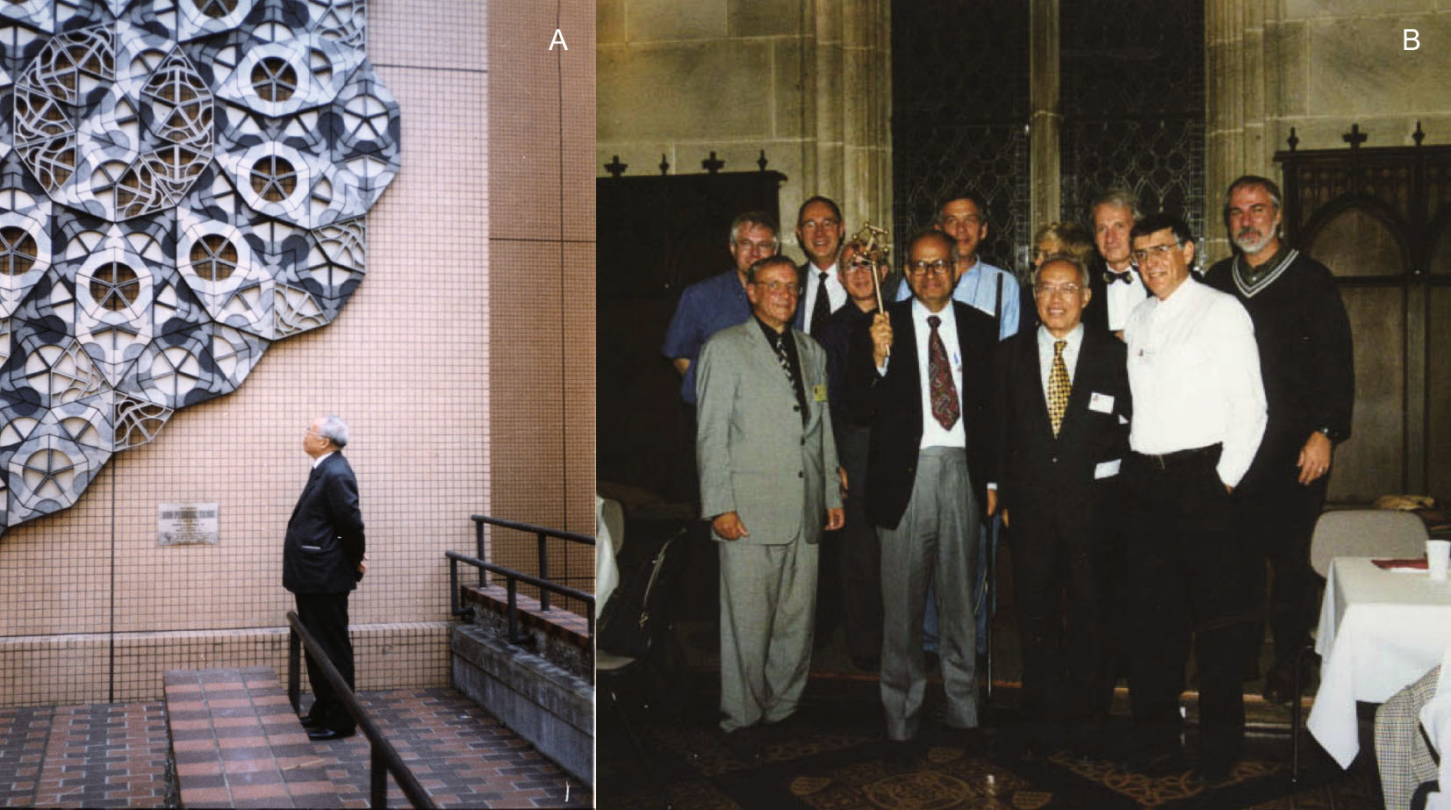
**In front of a Penrose tiling at the Tokyo Metropolitan University (A)**. **Attending the 7th International Conference on Quasicrystals, Stuttgart in 1999 (Front row, second from the right) (B)**

Professor Kuo quickly turned his full attention to the research of quasicrystals (Fig. 2). His contributions include (1) discovering more alloys where quasicrystals can exist and (2) developing an approximant method to approach the atomic structure of various quasicrystals (Kuo, 2004). Professor Kuo’s team discovered not only more three-dimensional quasicrystals with icosahedral symmetry, but also one- and two-dimensional quasicrystals with several other types of symmetries (Kuo, 2004). As one of the most productive and proficient centers in this field, his team was referred to as the “Kuo School” by the international quasicrystal community. Using his knowledge of the atomic packing in these topologically close-packed structures down to the fundamental antiprisms with a distorted icosahedral symmetry, he was able to establish many structural connections between the stable or metastable crystalline phases of a variety of alloys having the potential to form quasicrystalline structures. This allowed him to guide his students successfully to discover new quasicrystals in many unexplored metallic systems. In particular, icosahedral quasicrystals were subsequently identified in nearly twenty other Al- and Mn-based alloys. His group was also the first to discover a stable phase of two-dimensional decagonal quasicrystals of ten-fold symmetry in an Al-Co-Cu alloy, two-dimensional octahedral quasicrystals in Cr-Ni-Si and Mn-Si alloys, and dodecagonal quasicrystals with twelve-fold symmetry in Cr-Ni-Si and V-Ni-Si alloys. Additionally, one-dimensional quasicrystals were also discovered by his team in Al-Co-Cu and Al-Ni-Si alloys. With the accumulation of knowledge on such quasicrystals, he began to search for an eventual solution to the atomic structure of quasicrystals. While most scientists were fascinated with cutting-and-projecting higher dimensional cubic lattices in order to obtain structural models for quasicrystals, Professor Kuo took a new approach. Recognizing the proximity between the glassy and stable structures of the forming alloys, he was able to produce rational structures with quasicrystalline symmetry through minor shifts in the atomic positions. This method led to tremendous success in the study of quasicrystal structures, and it is now often referred to as the Kuo School’s approximant method.

In total Professor Kuo published more than 250 research papers; among them about 170 are on quasicrystals including one published posthumously (Cao & Kuo, 2008). His work on quasicrystals was well recognized, both domestically and internationally. He was frequently invited to speak at international conferences and was presented with numerous awards for his contributions to quasicrystal research, including the First Class National Science Medal in 1987 (with Heng-Qiang Ye, Douxing Li, Ze Zhang and Da-Neng Wang). In addition, he was also elected as an Honorary Member of both the Japanese Institute of Metals and the Materials Research Society of India.

## Efforts in bringing biological cryo-electron microscopy to China

In September of 1981, some twenty-five years after he left Europe and returned to China, Professor Kuo visited Sweden. During his one-month stay there, besides meeting with old friends and new, Professor Kuo was eager to learn what was at the forefront of electron microscopy and crystallography research. He was given a copy of the proceedings of the 47th Nobel Symposium: “Direct Imaging of Atoms in Crystals and Molecules” that took place in 1979. One particular article in the volume caught his attention: the one by Aaron Klug from the Molecular Biology Laboratory of the Medical Research Council, in which he described both his own work on the image reconstruction of T4 bacteriophage and the structure determination of bacteriorhodopsin from electron diffraction and imaging by Richard Henderson and Nigel Unwin (Klug, 1979). Professor Kuo kept the book in his laboratory at IMR, and encouraged everybody in the group to read it. At the same time, his group set up an optical diffractometer to examine image quality and to filter out noises. When the Nobel Prize in Chemistry was awarded to Klug in 1982, Professor Kuo became even more excited about macromolecular electron microscopy and image processing.

In an article entitled “Crystallographic electron microscopy and the Nobel Prize” that Professor Kuo published in the second issue of the 1983 *Journal of Chinese Electron Microscopy Society*, he described the new concept and technical advances in phase contrast, image processing and reconstruction, as well as their applications with various macromolecular complexes and viruses (Kuo, 1983). His thinking at the time was most clearly revealed by the last paragraph of the article: “Trained as a physicist, Dr. Klug works on biological macromolecules, but has received a Nobel Prize in Chemistry…Such (success) shows that physics, chemistry and biology exchange ideas and have got merged at this novel field called high resolution electron microscopy.” Without Klug’s deep knowledge in all three areas, “it would be hard to imagine that he could have accomplished so much … — this is a point that we all electron microscopists should ponder about.” In a book that he published in 1985, he again wrote extensively in the first chapter about the work of Klug, Henderson and Unwin and advocated for the great potential of the new field (Kuo and Ye, 1985).

Professor Kuo did not just reflect on it, this new field also spurred him into action. In early 1985 he sent a student to the University of Stockholm to learn image processing. This was followed over the years by his encouragement to more students to switch to structural biology including, Huilin Li, Dan Shi, Haixin Sui, Li Xing, Xing Zhang, Jinghua Tang, Donghua Chen, Garry Ren, Jianlin Lei, Yuan Wu, Binbin Deng and Juanfang Ruan, many of whom are still active in the cryo-EM field to this day (Li et al., 2003).

**Figure 3:**
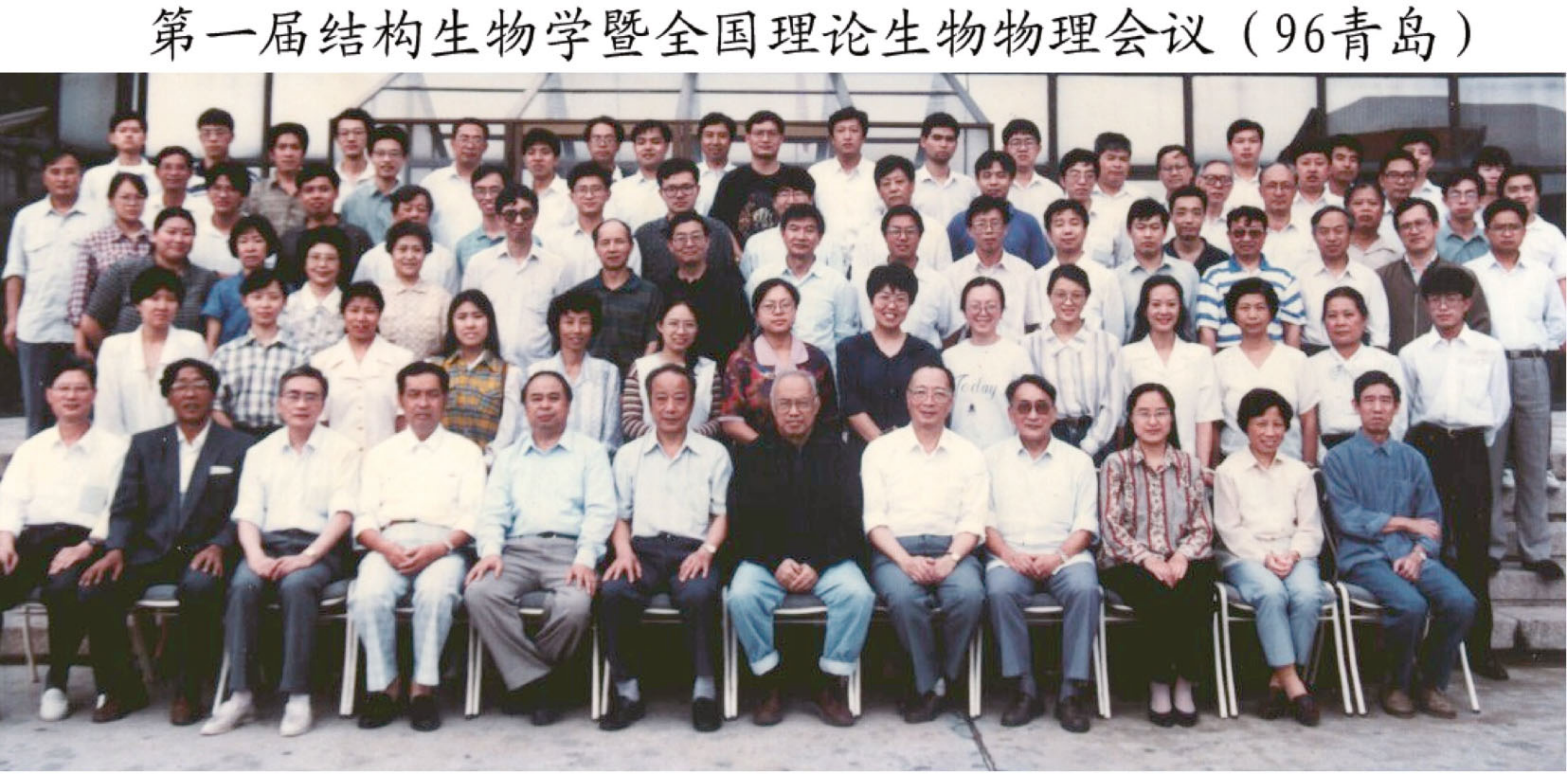
**Attending the First Chinese Conference of Structural Biology and Theoretical Biophysics, Qingdao in 1996 (Front row, 6th from the right**. Photograph courtesy of Dr. Haixu Tang, Indiana University)

Professor Kuo also wanted to start a biological cryo-electron microscopy group. His move to Beijing to establish the BLEM provided just such an opportunity (Fig. 3). He purchased a Gatan 626 cryo-stage and dedicated one electron microscope to biological research. In 1993, he recruited Wei Xu, a professor at the Institute of Biophysics who recently returned from Purdue University, as a group leader at BLEM, and assigned three of his own graduate students to work with Xu. Despite skepticism from some members of the local structural biology community about the potential of cryo-EM, Professor Kuo was able to obtain two research grants from the Chinese Natural Science Foundation to support projects on biological cryo-EM. Xu’s research there focused on structural studies of the plant light-harvesting complexes from two-dimensional crystals, and of icosahedral viruses, and the group was able to publish several papers (Zhang et al., 1997; Xu et al., 1998; Zheng et al., 2000; Zheng et al., 2001). To promote the field, Professor Kuo once invited Sen-Fang Sui, a professor at Tsinghua University, to join him at a meeting with the President of the Chinese Academy of Sciences in order to convince the Academy to invest more in the field. Professor Kuo also invited various investigators from overseas including Professor Wah Chiu of Baylor College, USA and Professor Wolfgang Baumeister of Max Planck Institute of Biochemistry, Germany to visit BLEM, and several of them including Da-Neng Wang, Z. Hong Zhou and Huilin Li spent a week or two there working with his students. Such early efforts planted the seeds for the future development of cryo-electron microscopy research in China.

## Training young scientists

Professor Kuo was a great mentor. He was very dedicated to the training of his students and ensuring their career success. Even years after they left his lab he would remain in touch with them to provide support and encouragement. From 1956 until the “Cultural Revolution” he trained several graduate students (Ye et al., 2014). After that dark period ended, students flocked to his laboratory at the IMR and later at BLEM. Between 1982 and 2000, he often accepted five to ten students per year. Over the years, he had trained close to 130 graduate students and approximately ten postdocs.

Professor Kuo trained his students with father-like love, combining Western scientific research methodology and traditional Eastern teaching philosophy. His passion for science was infectious. His impeccable taste in science and broad knowledge in metallurgy, chemistry, and crystallography allowed him to suggest great thesis projects. He told every entering class the story of the renowned German physicist Arnold Sommerfeld: legend said that Sommerfeld had a drawer in which a number of small pieces of paper were kept; each time a new student joined the lab he would open the drawer and give a slip to the student; a thesis project was written on the paper and the project often led to a Nobel Prize!

Once a thesis project was chosen, Professor Kuo’s students would need to search for and get daily help on things ranging from preparing specimens to recording high resolution electron micrographs from senior students or other lab members, not unlike in a traditional Chinese private school in the Confucius style. Such close interactions with each other made Professor Kuo’s students into a group of lifelong friends and collaborators. Professor Kuo himself would expect every student to deliver new experimental results regularly. He was well known for asking “What are your new results?” every time he ran into a student, sometimes once in the morning and once again in the afternoon. If a student did not get new results in a few weeks, he or she knew they would have to work harder.

Not only did he care about obtaining new results, he cared equally as much for the well-being of his students. Almost every student has some personal stories about Professor Kuo inviting him to his home for dinner, or buying his newborn daughter a gift, or receiving presents sent by him from China while working abroad. Four days before he passed away—right before he was sent to the intensive care unit—he penned a letter to a colleague saying that he no longer had the strength and could this colleague please help to revise a manuscript for a student.

**Figure 4:**
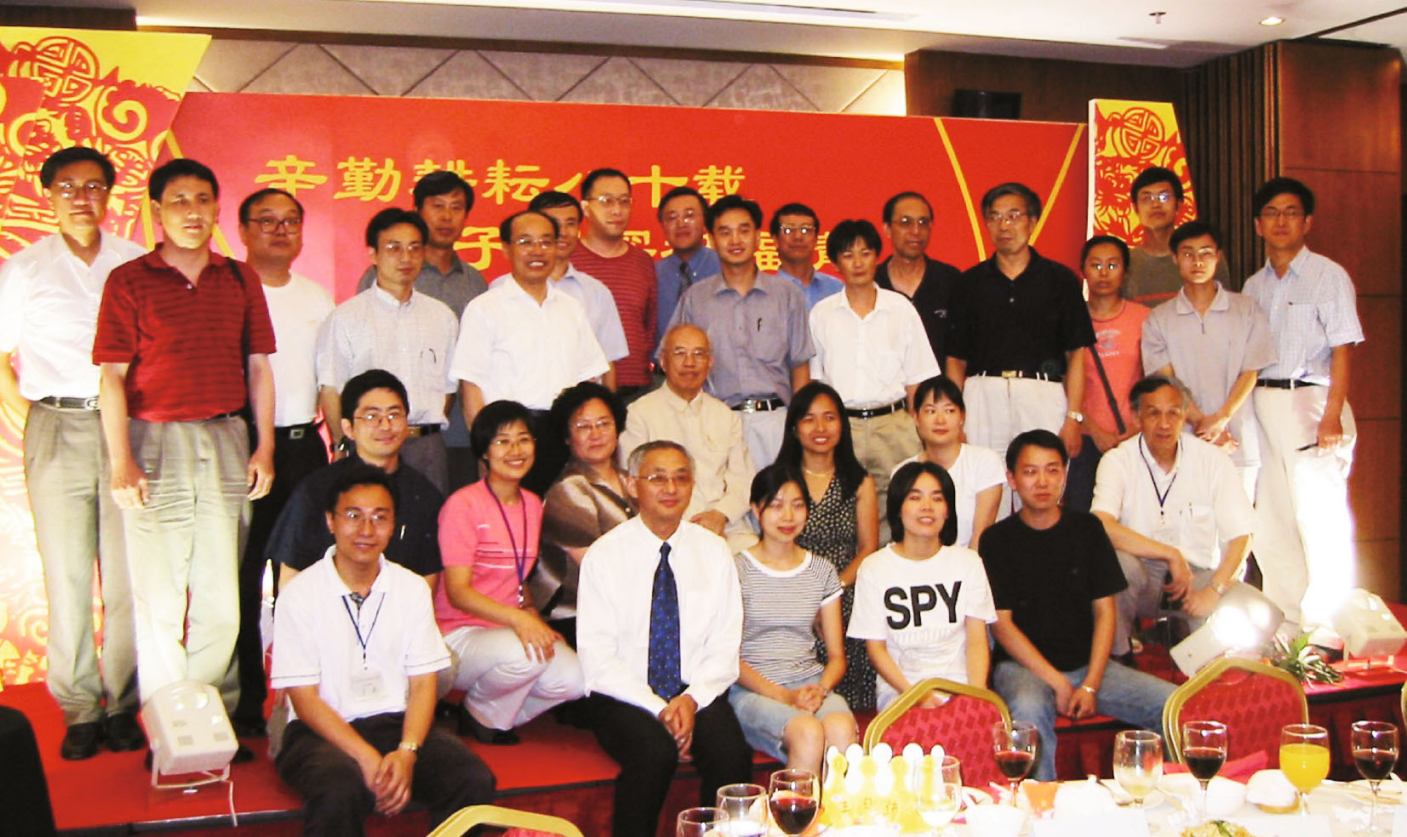
**With his former students who attended his 80th birthday celebration in Beijing, August 23rd, 2003 (Second row, center)**

Professor Kuo’s students did very well in his lab (Fig. 4) (Ye and Wang, 2003). The students were passionate about their projects, and they often worked long hours in the lab. In the early 1980s’ when the lab had only one electron microscope for high resolution imaging, it was usually kept manually running nonstop for 24 h a day, with students working at the microscope in six-hour shifts. Professor Kuo directed the students to exciting areas, and they often discovered something new. Almost without exception, everybody was able to publish their thesis work in international top journals of physics or materials science—very uncommon for a laboratory in China in the 1980’s and 1990’s—some even published as many as 15 papers!

After training with Professor Kuo and starting their own independent careers, many of his former students accomplished a great deal in research, including three who became academicians of the Chinese Academy of Sciences: Heng-Qiang Ye, Ze Zhang, and Lijun Wan. Several of his former students also won international acclaim for their achievements in science and have received prestigious prizes: Xiaodong Zou was awarded the 2008 Göran Gustafsson Prize in Chemistry by the Royal Swedish Academy of Sciences and was elected to be a member of the Royal Swedish Academy of Engineering in 2017, and in 2014 Chunlin Jia received the H. Hashimoto Medal from the International Federation of Societies for Microscopy.

## Leadership and legacies

In addition to founding and directing two major electron microscopy laboratories in Shenyang and later in Beijing, as a senior figure in the science and engineering community in China, Professor Kuo also devoted much of his energy to the establishment and development of the Chinese electron microscopy community (Ye et al., 2014). Two years after the founding of the Chinese Electron Microscopy Society (CEMS), he became its President in 1982 and served in this office until 1996. It was during this period that Professor Kuo was instrumental in re-establishing connections with the international community, including his efforts and success in making CEMS a member of the International Federation of the Societies of Electron Microscopy (IFSEM). He also served as President of the Federation of Asia-Pacific Societies for Electron Microscopy (1993–1996) and as a member of various international science committees and on the editorial boards of numerous professional journals. He organized the Fifth Asia-Pacific Electron Microscopy Conference in Beijing in 1992, the International Union of Crystallography (IUCr) Congress Satellite Meeting on quasicrystals in Beijing in 1993, and initiated the annual Sino-Japanese Bilateral Electron Microscopy Symposia, which helped tremendously to establish connections between the Chinese electron microscopists and the international community. As President of the CEMS, he solicited financial support to allow more scientists to participate and present results at international conferences during the time when international travel funds were scarce in China. As a result, noticeable Chinese attendance was made at the IFSEM Conferences in Kyoto (1986) and in Seattle (1990).

To celebrate Professor Kuo’s contributions to science and his dedication to the training of young scientists, his students and friends established the K.H. Kuo Education Fund, a non-profit organization to promote advances in electron microscopy. In collaboration with the Chinese Electron Microscopy Society, the K.H. Kuo Education Fund has awarded the K.H. Kuo Distinguished Scientist Award and the K.H. Kuo Young Scientist Award biennially since 2002.

Professor Kuo passed away on December 13th, 2006. We were all saddened by the loss of a great mentor, a father-like advisor and a dear friend. As a memorial to honor his contributions to electron microscopy, a summer school series, the K.H. Kuo Summer School of Electron Microscopy and Crystallography, was launched in 2008 with the aim of bringing leading scientists to China to interact with young scientists, as Professor Kuo did for us over 35 years ago. The summer school is held annually and the topic alternates between structural biology and materials science. Over the years, the school series has become one of the most prestigious and influential meetings in electron microscopy (Table 1). The first summer school, held in 2008, was attended by fewer than one hundred people; the 11th school in 2018 attracted about four hundred participants, a quarter of them coming from abroad; schools for the next three or four years have already been planned. Indeed, over the years the school series has been able to attract the very top scientists to attend and lecture. For example, Professor Joachim Frank, a winner of the 2017 Nobel Prize in Chemistry for developing single-particle cryo-EM, has lectured in four of the six biological Kuo summer schools so far, whereas Dr. Richard Henderson, his co-winner, has attended twice. This school series, along with the passion for science and the efforts of the people who were trained with or influenced by him, will help keep Professor Kuo’s legacy alive.

**Table 1:** **K. H. Kuo Summer School of Electron Microscopy and Crystallography.**

Year	Field	Chairs	Host	Location
1st/2008	Cryo-EM of macro-molecular complexes	Sen-Fang Sui Da-Neng Wang	Tsinghua University	Beijing
2nd/2009	Materials science	Ze Zhang Lu-Chang Qin	Zhengzhou University	Zhengzhou
3rd/2010	Cryo-EM of macro-molecular complexes	Fei Sun Da-Neng Wang	Institute of Biophysics, Chinese Academy of Sciences	Beijing
4th/2011	Materials science	Xiuliang Ma Xiaodong Zou	Institute of Metal Research, Chinese Academy of Sciences	Shenyang
5th/2012	Cryo-EM of macro-molecular complexes	Z. Hong Zhou Gang Cai Guoqiang Bi	University of Science and Technology of China	Hefei/Huangshan
6th/2013	Materials science	Jin-Ping Zhang	Suzhou Institute of Nano-Tech and Nano-Bionics, Chinese Academy of Sciences	Suzhou
7th/2014	Cryo-EM of macro-molecular complexes	Huilin Li Yifan Cheng Yao Cong Yongning He	National Center for Protein Science	Shanghai
8th/2015	Materials science	Ze Zhang	Zhejiang University	Hangzhou
9th/2016	Cryo-EM of macro-molecular complexes	Hongwei Wang Peijun Zhang	Tsinghua University	Beijing/Huairou
10th/2017	Materials science	Zhiwei Shan	Xi’an Jiaotong University	Xi’an
11th/2018	Cryo-EM of macro-molecular complexes	Xing Zhang Jun Liu Haixin Sui	Zhejiang University	Hangzhou
12th/2019 (planned)	Materials science	Xiaodong Han Jin Zou	Beijing University of Technology	Beijing
13th/2020 (planned)	Cryo-EM of macro-molecular complexes	Qingtao Shen Wen Jiang	Shanghai University of Science and Technology	Shanghai
15th/2022 (planned)	Cryo-EM of macro-molecular complexes	Peiyi Wang Xiaochen Bai	Southern University of Science and Technology	Shenzhen

Professor Ke Hsin Kuo’s insightful scientific vision, eclectic taste of research themes, and his warm, generous and engaging personality, have left us with great memories and sincere gratitude. He will be remembered as a dear teacher, a great mentor and a devoted scientist, whose character is best described by the motto that he himself cherished dearly, *Live in Immaculacy*; *Work with Dedication* (清清白白做人,认认真真做学问).
